# Effects of Dietary Net Energy/Lysine Ratio and Sex on Growth Performance, Digestive Organ Development, and Cecal Microbiota of Broiler Chickens

**DOI:** 10.3390/ani15111572

**Published:** 2025-05-28

**Authors:** Zhibin Ban, Simiao Chen, Lijia Li, Qiyu Zhang, Xiaodong Zhao, Hao Liang, Yuming Guo

**Affiliations:** 1 State Key Laboratory of Animal Nutrition and Feeding, College of Animal Science and Technology, China Agricultural University, Beijing 100193, China; banzb0620@163.com; 2 Jilin Academy of Agriculture Sciences (Northeast Agricultural Research Center of China), Gongzhuling 136100, China; chensm1999@163.com (S.C.); 18704492374@163.com (L.L.); zhangqiyu1130@163.com (Q.Z.); z13843423030@163.com (X.Z.); l13596639477@163.com (H.L.)

**Keywords:** male and female broilers, net energy level, lysine level

## Abstract

This study aimed to investigate the effects of the net energy (NE) and lysine ratio in low-protein diets on growth performance, intestinal development, and cecal microbiota of male and female broilers. A 4 × 2 factorial design was used with lysine levels at 1% and 1.5%, and net energy levels at 8.93 MJ/kg and 9.76 MJ/kg were used to form four diets with net energy/lysine ratios: Group I (8.93), II (5.95), III (9.76), and IV (6.50), respectively. A total of 960 AA broilers at age of 1 d were selected; then, 480 male and 480 female broilers were randomly divided into four groups, with eight replicates per group and 15 birds per replicate. The trial lasted for 17 days, with slaughter tests conducted separately at d 7 and 17 to measure growth performance and slaughter performance. The results are as follows: (1) At d 17, broilers in high NE/lysine groups had significantly higher final weights and average daily gain compared to other groups (*p* < 0.01), with males weighing more than females. (2) High NE/lysine ratios (8.93 and 9.76) significantly increased the relative lengths of the jejunum and ileum from d 1 to 17 (*p* < 0.05). At d 7, female broilers had greater relative lengths of the duodenum, jejunum, and ileum compared to males (*p* < 0.05, *p* < 0.05, and *p* < 0.01), while at d 17, male broilers had greater relative lengths of the duodenum and ileum than females (*p* < 0.01 and *p* < 0.05). (3) At d 7 and 17, the villus height to crypt depth ratio in male broilers was significantly lower than that in females (*p* < 0.05). There was an interaction effect between NE/lysine ratios and sex on intestinal morphology. (4) High NE/lysine ratios (8.93 and 9.76) resulted in higher levels of Firmicutes and Bacteroidetes. Male broilers had higher levels of Firmicutes and Verrucomicrobia compared to females. Therefore, when lysine was at an appropriate level, a high NE/lysine ratio was more conducive to the growth and development of broilers through improving intestinal development and microbiota abundance. Female broilers showed faster intestinal development at the early age but weaker absorption capacity, while males showed dominance in intestinal length development. There were differences in characteristic gut microbiota between male and female broilers, with males having a higher abundance of energy metabolism-related microbiota.

## Simple Summary

Amino acids and energy are the two most important factors in the diet of broilers. The digestion and utilization efficiency of nutrients are different between male and female broilers. The evaluation of the digestion differences between male and female broilers is helpful to provide a basis for precision nutrition. In this study, the effects of lysine/net energy on the growth and development of male and female broilers were investigated. The results show that, when the lysine level was appropriate, a high NE/lysine ratio was more beneficial to the growth and development of broilers by improving gut development and microbiota abundance, and there were differences in gut development and gut abundance between male and female broilers. The results are expected to provide a basis for breeding male and female broilers and optimizing feed formulation.

## 1. Introduction

The goal of poultry nutrition is to adjust the feed according to the actual production needs of broilers to maximize profitability. With limited protein resources and the continuous expansion of broiler farming, the demand for precise nutrition farming is becoming urgent. The key to precision nutrition is accurately matching the nutrient supply in the feed with the nutrient needs of animals, aiming to reduce feed waste and maximize the production potential of animals. The energy and amino acid levels in diets are critical factors in poultry production. Currently, the metabolizable energy (ME) system is widely used, but it tends to overestimate the effective energy value of proteins and underestimate the energy value of fats. Compared to the ME system, the net energy (NE) system accounts for the heat increment from feed intake and digestion, thus more accurately reflecting the true energy requirements of animals [1,2]. Lysine, as the second limiting amino acid for broilers, plays a crucial role in growth and physiological metabolism. At an appropriate level, lysine can significantly enhance growth performance, carcass traits, and promote cecal microbiota diversity [3,4]. However, deficient or excess lysine can inhibit protein synthesis and lymphocyte proliferation, impairing growth and potentially causing toxicity [5]. Studies have shown that there are differences in growth performance, body weight, nutrient digestibility, gene expression of nutrient transport proteins, and gut microbiota between male and female broilers [6,7]. Hernandez et al. [8] fed control diets with crude protein (CP) levels of 24.5%, 23.0%, 21.5%, and 20.5% in four stages, and provided medium- and low-CP treatments where protein levels were 1.5% and 3% lower than the control. The results showed that, during these four stages, reducing CP by up to 3% had no effect on the production performance of females. However, in male broilers, a reduction in dietary CP levels negatively affected performance. These results confirmed the differences in protein requirement between sexes, showing that female broilers may have lower protein needs than males. Feeding male and female broilers separately is an effective method to control body weight, improve survival rates, and enhance the feed conversion ratio based on their different nutritional requirements. Most current research focuses on the effects of metabolizable energy and protein levels on mixed-sex broilers, while there is limited research on the effects of the net energy/lysine ratio on male and female broilers [9]. Therefore, this study aimed to investigate the effects of dietary net energy/lysine ratios on the growth performance, intestinal development, and cecal microbiota of broilers of different sexes and to compare the growth and development differences between male and female broilers, providing data and theoretical support for precision feeding in modern broiler production.

## 2. Materials and Methods

### 2.1. Ethics Statement

The study was approved by the Animal Ethics Committee of the Jilin Academy of Agricultural Sciences, and all procedures were conducted in accordance with the protocol approved by the Science Ethics (Review) Committee of the Jilin Academy of Agricultural Sciences (Northeast Agriculture Research Center of China), under approval number JNK20230810-1.

### 2.2. Experimental Design and Animal Husbandry

The experiment used a 4 × 2 factorial design (Table 1). Lysine levels were set at 1% and 1.5%, with NE levels at 8.93 MJ/kg and 9.76 MJ/kg. The calculated net energy/lysine ratios were Group I (8.93), Group II (5.95), Group III (9.76), and Group IV (6.50). A total of 960 AA broilers (480 males and 480 females) with similar body weights were selected. Then, the male and female broilers were randomly assigned to four groups, with eight replicates per group and 15 birds per replicate. The experiment lasted 17 days. Slaughter tests were conducted on d 7 and 17 to measure growth and slaughter performance.

### 2.3. Experimental Diets

The experimental diets were all low-protein diets, with protein levels reduced by 3% according to the “AA Broiler Management Handbook 2019”. The protein level was maintained at 20%. The corn–soybean meal diet was chosen and pelleted at all age stages. The composition and nutrient levels of the diets are shown in Table 2. The net energy values of the test diets were derived from the Poultry Net Energy Formula software (2022SR0482429), jointly developed by China Agricultural University and the Jilin Academy of Agricultural Sciences. The actual net energy values were measured using indirect calorimetry at the Joint Research Center for Poultry Energy Nutrition, co-built by China Agricultural University and the Jilin Academy of Agricultural Sciences. A 12-chamber poultry open-circuit respiratory device was used for AA broilers aged d 14–17, with six replicates per diet and three chickens per replicate. The results are 8.93 MJ/kg and 9.76 MJ/kg.

### 2.4. Measured Indicators and Methods

#### 2.4.1. Growth Performance

Broilers were weighed at d 1, 7, and 17. During the experiment, feed consumption per replicate was recorded to calculate average daily gain (ADBG), average daily feed intake (ADFI), and feed conversion ratio (F/G).

Average Daily Body Gain (ADG) = Total Weight Gain/(Number of Chickens × Days)

Average Daily Feed Intake (ADFI) = Total Feed Consumption/(Number of Chickens × Days)

Feed Conversion Ratio (F/G) = Total Feed Consumption/Total Weight Gain

#### 2.4.2. Intestinal Development

At d 7 and 17, two chickens close to the average body weight from each replicate were selected, weighed, and then euthanized by neck vein bleeding. The ileum, duodenum, and jejunum were dissected, and excess fat surrounding the intestines was removed. The lengths of each intestinal segment were measured with a tape measure.

#### 2.4.3. Intestinal Morphology

A 2 cm section from the middle of the jejunum was taken, flushed with saline, and fixed in 10% paraformaldehyde. The samples were processed by Wuhan Servicebio Biological Co. (Jilin, China), dehydrated, cleared, embedded in paraffin, sectioned, and stained with hematoxylin and eosin (HE). The villus height (VH) and crypt depth (CD) were observed under a microscope, and villus-to-crypt ratio (VH/CD) was calculated.

#### 2.4.4. Cecal Microbiota

The v3–v4 regions of the 16S rRNA gene were amplified, using the forward primers 341f and the reverse primer 805r separately. Each experimental group for each sex included 6 biological replicates (e.g., n = 6 male-I per group). Sequencing was conducted at the Genome Center of Shanghai Paiseno Biotechnology Co., Ltd. (Shanghai, China) The Illumina platform was used for the paired-end sequencing of community DNA fragments. The raw sequencing data were saved in the FASTQ format. DADA2 was used for sequence denoising to obtain amplicon sequence variants (ASVs), which provide higher taxonomic resolution than traditional operational taxonomic units (OTUs). Alpha diversity indices (Chao, Shannon, Simpson, and Faith) were analyzed using Dunn’s test for post hoc comparisons to verify significance. To further compare species composition differences between samples, petal diagrams were used for analyzing the community, and heat maps were constructed to analyze species composition through using the abundance data of the top 20 genera.

### 2.5. Data Analysis

Experimental data were analyzed using a two-way ANOVA in the SPSS 27.0 software. Results are expressed as means, and Duncan’s method was used for multiple comparisons. Differences were considered significant at *p* < 0.05 and extremely significant at *p* < 0.01.

## 3. Results

### 3.1. Growth Performance

The effects of different NE/lysine ratios and sex on the growth performance of broilers at d 1–7 and d 1–17 are shown in Table 3. At d 7, the NE/lysine ratio had no significant effect on male and female broilers, and there were no significant differences in development between the sexes. At d 17, broilers in the high NE/lysine groups (8.93 and 9.76) had significantly higher final body weights and average daily gain compared to the other groups (*p* < 0.01). The body weights of males were significantly higher than those of females (*p* < 0.05).

### 3.2. Intestinal Development

The effects of different NE/lysine ratios and sex on digestive organ development in broilers at d 1–7 and d 1–17 are shown in Table 4. It shows that the NE/lysine ratio had no significant effect on the relative development of digestive organs in broilers from d 1 to 7. However, high NE/lysine ratios (8.93 and 9.76) significantly increased the relative lengths of the jejunum and ileum from d 1 to 17 (*p* < 0.05). At d 7, the relative lengths of the duodenum, jejunum, and ileum in females were significantly higher than those in males (*p* < 0.05, *p* < 0.05, and *p* < 0.01), while at d 17, the relative lengths of the duodenum and ileum in males were significantly higher than those in females (*p* < 0.01 and *p* < 0.05).

### 3.3. Intestinal Morphology

**Figure 1:**
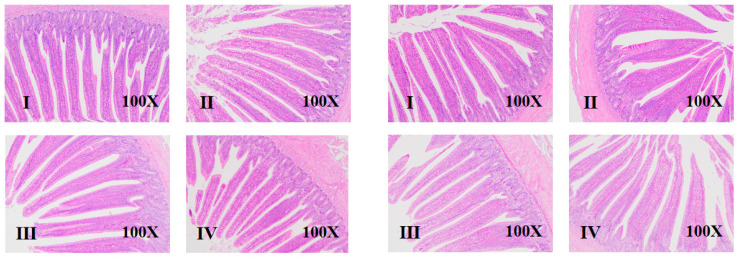
Effects of net energy/lysine and sex on the intestinal morphology of broilers at d 7 (Four groups on the left are male broilers, and four groups on the right are female broilers. I–IV means group I–IV).

**Figure 2:**
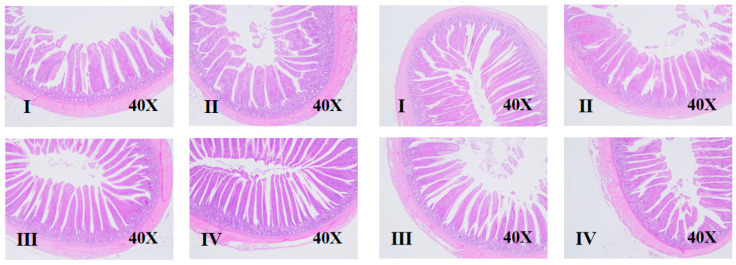
Effects of net energy/lysine and sex on the intestinal morphology of broilers at d 17 (Four groups on the left are male broilers, and four groups on the right are female broilers. I–IV means group I–IV).

The effects of different NE/lysine ratios and sex on intestinal morphology in broilers at d 1–7 and d 1–17 are shown in Table 5 and Figure 1 and Figure 2. Different NE/lysine ratios had no significant effect on the intestinal morphology of male and female broilers. However, at d 7 and 17, the villus height-to-crypt depth ratio (VH/CD) in males was significantly lower than that in females (*p* < 0.05). There was an interaction between NE/lysine ratios and sex on the intestinal morphology of broilers.

### 3.4. Cecal Microbiota

**Figure 3:**
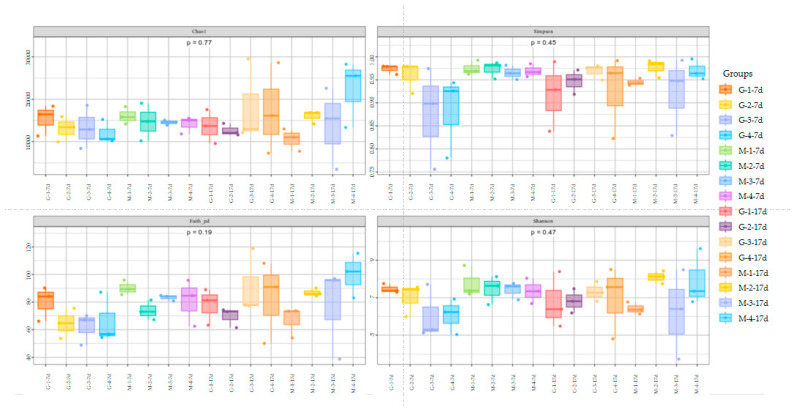
Alpha diversity of cecal microbiota in male and female broiler chickens at d 7 and 17 (G: male, M: Female).

**Figure 4:**
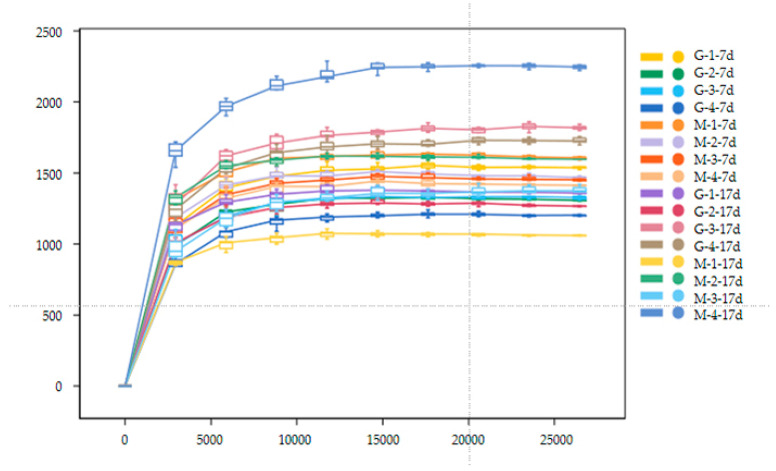
Rarefaction curves of cecal microbial communities in male and female broiler chickens at d 7 and 17 (G: Male, M: Female).

**Figure 5:**
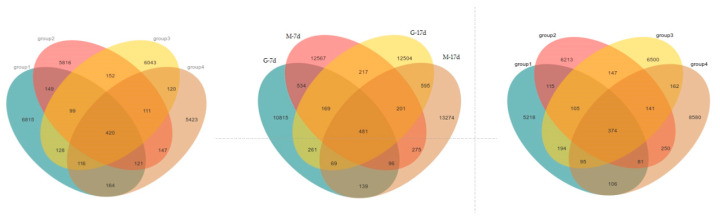
Venn diagrams of cecal microbial communities of male and female broilers at d 7 and 17. Note: the left figure is the Venn diagram for broilers at d 7; the middle figure is the Venn diagram for broilers at d 17; and the right figure is the Venn diagram of male and female broilers at different ages.

**Figure 6:**
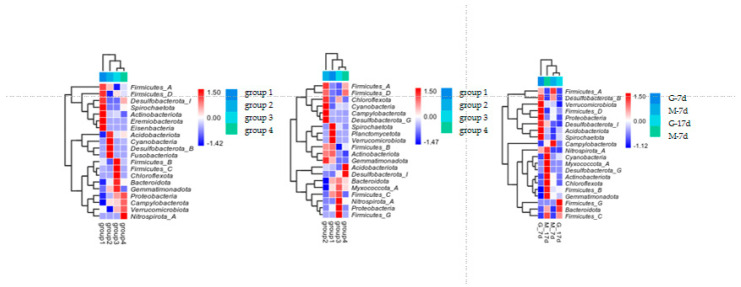
Heat map of cecal contents of male and female broilers at d 7 and 17. Note: the left picture shows the heat map of four groups at d 7; the middle picture shows the heat map of four groups at d 17; and the right picture shows the heat map of male and female broilers at different ages.

Figure 3 shows the alpha diversity and rarefaction curves of the cecal microbiota in the contents of male and female broilers at d 7 and 17. Alpha diversity indices (Chao1, Shannon, etc.) showed no statistically significant differences among groups at the genus level (*p* > 0.05, Dunn’s test), suggesting comparable overall microbial diversity across treatments. When the number of sequences reached 25,000, the rarefaction curves for the observed species became flat, indicating a sufficient sequencing depth (Figure 4). The Venn analysis of the number of ASVs in the samples (as shown in Figure 5) showed that there were 420 core ASVs shared among the four treatment groups (Groups I–IV) at d 7 and 374 core ASVs at d 17. There were 1280 core ASVs shared between male and female broilers at d 7 and 1346 core ASVs at d 17. The heat map results indicate that Group I and Group III had higher contents of Firmicutes and Bacteroidetes, and males had higher contents of Firmicutes and Verrucomicrobia compared to females (as shown in Figure 6). The SPSS analysis of the data is shown in Table 6 and Table 7. Statistically, the content of Firmicutes in males was higher than that in females at d 7 (*p* = 0.017), but there was no significant difference at d 17, and there was no significant difference in cecal flora under the influence of different groups.

## 4. Discussion

Amino acids and energy are important components of poultry diets, but it does not mean that the higher, the better. The relative balance of various amino acids in the diet is more important; the better the balance of essential amino acids in the diet, the higher the utilization rate of dietary protein by animals [10]. The same applies to energy levels; excessively high energy levels in the diet may lead to fat deposition. Some studies have pointed out that reducing the energy level of broiler feed by about 1 MJ/kg can significantly reduce abdominal fat rate and body fat deposition without adversely affecting final live weight, average daily gain, feed intake, breast muscle yield, or slaughter rate [11,12]. Therefore, exploring the appropriate lysine energy ratio is beneficial for the development of the broiler industry. Both energy and amino acid levels in the diet affect feed intake; poultry essentially eat for energy. Taylor and Kyriazakis pointed out that, after reducing dietary energy levels, poultry increase feed intake to stabilize body energy [13]. Barekatain et al. explored the effects of digestible amino acids on broilers and found that, when the AME content was similar, broilers might consume more feed that better balances amino acids and energy [14]. The results in Table 3 show that there was no significant difference in feed intake among groups in this study, indicating that the effect of lysine energy level on broilers was not related to intake but to the nutrient utilization rate [15]. Comparing the lysine and net energy levels among the groups, it was found that the same lysine level with different energy levels had no significant effect on broiler growth performance. However, after controlling the net energy level, increasing lysine from 1.0% to 1.5% significantly reduced the final body weight of broilers. Therefore, the differences in growth performance in this study may have resulted from excessive lysine, causing antagonistic effects with other amino acids, leading to amino acid imbalance and reduced nutrient absorption and utilization. Barekatain’s experimental results indicated that the effects of digestible lysine and energy levels on feed consumption and body weight gain seemed primarily independent, and the independent responses of feed intake and weight gain were consistent with the experimental hypothesis, similar to the results of this study [14]. Meanwhile, the results of this study show that the final body weight of male broilers at d 17 was significantly higher than that of females, which is consistent with most current research findings [16,17]. Lopez et al. studied the effects of sex on broiler final body weight and found significant differences in final body weight between male and female broilers at d 42, with males being heavier than females [7]. Goo et al. found that, although male broilers were heavier, the coefficient of variation (CV) was higher [18]. In experiments, the CV should be kept as low as possible because increased body weight variability may affect the overall results of the experiment, and the goal of the experiment is to keep unnecessary changes to a minimum. Madilindi et al. pointed out that, although there were differences in body weight at various stages of broiler growth, the feed conversion ratio (FCR) was generally the same, and they showed the same degree at the same age, which is also consistent with the results of this experiment [17].

The small intestine is the primary site for the digestion and absorption of nutrients, and its developmental speed is a key factor in ensuring the rapid growth of broilers [19]. Studies have shown that newly hatched chicks have a small digestive tract volume (especially the stomach) and weak gizzard grinding capacity, but within 10 days, they rapidly develop in terms of morphology and function. The relative weights of various organs reach their peak within d 7–10, with the peak times being: gizzard at d 4–5, pancreas at d 9, and small intestine at d 7–10 [10]. A well-developed small intestine structure and function are fundamental for animals to digest and absorb nutrients and grow healthily [19]. The villi in the small intestine are the primary sites for nutrient absorption in the intestinal cavity. The longer or higher the villi, the larger the surface area available for nutrient absorption and the stronger the absorption capacity. Crypts are tubular structures between the bases of the villi and the submucosa, mainly composed of undifferentiated cells. Deeper crypts indicate more undifferentiated cells and less mature intestinal development, while shallower crypts indicate more mature intestinal development, with stronger secretion and absorption capabilities as age increases [20,21]. Griela et al. pointed out that, as dietary energy and protein intake decrease, the overall antioxidant response of the broiler chicken gut increases [22]. However, Adewole et al. pointed out that high-energy-density diets may increase body weight at the expense of gut health [23]. In this study, Table 4 and Table 5 show the relative weight of digestive organs and intestinal morphology development results, respectively. The results indicate that the high NE/lysine ratios (8.93 and 9.76) in Groups I and III significantly increased the relative lengths of the jejunum and ileum in 17-day-old broilers (*p* < 0.05). This trend is consistent with growth performance, where high NE/lysine ratios promoted broiler growth and development through improved intestinal function. The results also show significant differences between male and female broilers at all age stages, displaying sex-specific characteristics. First, the intestinal morphology development in females was better than that in males at both stages, but the relative weight of digestive organs showed opposite results at different ages: females developed faster at d 7, while males developed faster at d 17. Combining these findings, it appears that the rapid intestinal development in males may be one of the main reasons for their rapid body weight growth. However, combining growth data, it was found that growth performance in females did not change at d 7, while at d 17, growth performance in males significantly improved, suggesting that, for males, the relative length of the intestine plays a dominant role in nutrient absorption. Although females showed a faster early intestinal length development, their absorption capacity was weak, which may be related to the secretion timing of digestive enzymes. The primary secretion of digestive enzymes peaks around d 7–10, with pancreatic amylase, trypsin, chymotrypsin, and pancreatic lipase activities peaking at d 10, 7, and 21, respectively, and the intestinal amylase, trypsin, chymotrypsin, and lipase activities all peaking at d 10 [24]. They pointed out that the activity of intestinal mucosal enzymes increases with age and plays a rate-determining role in providing growth substrates, which also indicates that broiler intestinal development is not a single independent process but involves a coordinated effort of relative length, morphology, and energy supply indicators.

The gastrointestinal microbiota is a complex community mainly composed of bacteria, containing various types of microorganisms. Our findings reveal consistent phylum-level dominance patterns across experimental groups: Firmicutes and Bacteroidetes collectively constituted 83–92% of the cecal microbiota (Figure 6), aligning with previous reports in poultry gut microbiomes [25]. This diverse population provides the host with a wide range of enzymes and substrates, which, combined with the host’s metabolic capabilities, offer a broad metabolic repertoire for nutrient and energy collection [26]; meanwhile, metagenomic studies have shown that the enrichment in glycoside hydrolase genes in these phyla endows them with the ability to degrade recalcitrant dietary polysaccharides [27]. According to the heat map analysis, the dominant bacteria in Groups I and III were dominated by Firmicutes regardless of age stage, which was significantly higher in males than in hens at 7 days of age after SPSS 27.0 calculation. Firmicutes is one of the important phyla in the growth of broilers. They ferment dietary fibers to produce short-chain fatty acids (SCFAs) such as acetate, propionate, and butyrate, which play a role in regulating gut health [28,29,30]. Our observation of higher Firmicutes/Bacteroidetes (F/B) ratios in these groups coincides with their superior growth performance. While the F/B-obesity association is well-established in mammals [31], our study extends this paradigm to poultry production systems. Yang showed that the abundance of Firmicutes was significantly positively correlated with feed efficiency (r = 0.42, *p* < 0.05), suggesting that the gut microbiota group plays a key role in broiler performance [32]. The sex-specific microbiota differences present intriguing implications for precision husbandry. Methodologically, our phylum-level focus provides robust biomarkers for industrial applications. While genus-level resolution offers taxonomic precision, phylum abundance profiles exhibit higher reproducibility across sequencing platforms (CV < 15% vs. 25–40% at genus level) [33], making them more suitable for large-scale monitoring. Future integration with metatranscriptomics could bridge the gap between community structure and functional outputs [34,35].

## 5. Conclusions

This study established an evidence-based framework for sex-specific net energy (NE)/lysine ratio optimization in broiler diets. When lysine was at an appropriate level, a high NE/lysine ratio was more conducive to the growth and development of broilers through improving intestinal development and microbiota abundance. Female broilers showed faster intestinal development at the early age but a weaker absorption capacity, while males showed dominance in intestinal length development. It reveals developmental dyssynchrony between gut morphology and microbial colonization patterns across genders. It can provide a reference basis for the precision breeding of male and female broilers.

## Figures and Tables

**Table 1:** Experiment design.

Item	NE Level MJ/kg	Lys Level%	NE/Lys
Group I	8.93	1.0	8.93
Group II	8.93	1.5	5.95
Group III	9.76	1.0	9.76
Group IV	9.76	1.5	6.51

**Table 2:** Composition and nutrient levels of the diets.

Item	Group			
	I	II	III	IV
Corn	65.33	62.24	60.91	60.91
Soybean meal	27.34	27.06	27.57	26.23
Soybean oil	0.90	2.80	4.71	4.71
Domestic extruded soybean	2	2	2	2
Corn gluten meal	0.56	0.73	0.90	0.90
NaHCO_3_	1.50	1.51	1.52	1.52
Limestone	1.43	1.43	1.43	1.43
NaCl	0.25	0.25	0.25	0.25
L-lysine hydrochloride	0.10	0.75	0.12	0.80
DL-Met	0.25	0.50	0.24	0.50
Salinomycin	0.05	0.05	0.05	0.05
Premix ^1^	0.20	0.20	0.20	0.20
L-Thr	0.09	0.48	0.10	0.50
Total	100	100	100	100
Nutrient level ^2^				
Poultry ME (MJ/kg)	12.45	12.45	13.28	13.28
Poultry NE (MJ/kg)	8.93	8.93	9.76	9.76
Crude protein (%)	21.50	21.50	21.50	21.50
Ca (%)	0.95	0.95	0.96	0.95
Non-phytic acid phosphor (%)	0.33	0.33	0.33	0.33
Lys (%)	1	1.50	1	1.50
Met (%)	0.53	0.78	0.52	0.77
Met + Cys (%)	0.84	1.08	0.83	1.07
Try (%)	0.20	0.19	0.20	0.19
Ile (%)	0.76	0.75	0.77	0.74
Arg (%)	1.19	1.18	1.19	1.14
Thr (%)	0.82	1.19	0.83	1.19
Dlys (%)	0.87	1.30	0.87	1.31
DMet (%)	0.48	0.71	0.47	0.70
DMet + DCys (%)	0.76	1	0.74	0.99
DThr (%)	0.74	1.07	0.75	1.06
DTry (%)	0.18	0.17	0.18	0.17
DArg (%)	1.09	1.08	1.09	1.05

Notes: ^1^ The premix provided the following per kg of diets: VA 12,500 IU, VB_1_ 0.01 mg, VB_2_ 8.00 mg, VB_6_ 4.5 mg, VB_12_ 0.02 mg, VD_3_ 3500 IU, VE 20 IU, VK_3_ 3 mg, biotin 0.2 mg, folic acid 0.5 mg, D-pantothenic acid 12 mg, nicotinic acid 34 mg, Cu (as copper sulfate) 8 mg, Fe (as ferrous sulfate) 80 mg, Mn (as manganese sulfate) 80 mg, Zn (as zinc sulfate) 80 mg, I (as potassium iodide) 0.70 mg, and Se (as sodium selenite) 0.30 mg. ^2^ Nutrient levels were measured values.

**Table 3:** Effects of net energy/lysine and sex on the growth performance of broilers at d 7 and 17.

NE/Lys	Sex	IABW/g	1–7 d				1–17 d			
			FABW/g	FCR	ADGW/g	ADFI/g	FABW/g	FCR	ADGW/g	ADFI/g
I (8.93)	Male	42.20	200.67	0.92	22.64	20.84	707.33	1.20	39.12	46.89
	Female	41.50	197.33	0.91	22.22	20.22	684.67	1.16	37.83	43.64
II (5.93)	Male	41.62	195.67	0.94	22.07	20.75	661.00	1.21	36.43	44.04
	Female	42.25	192.00	0.98	21.31	20.88	651.67	1.31	35.85	47.06
III (9.76)	Male	42.35	194.33	0.99	21.80	21.58	700.67	1.19	38.72	45.96
	Female	42.42	197.00	0.93	22.15	20.60	682.67	1.13	37.66	42.73
IV (6.50)	Male	41.50	195.67	0.93	22.04	20.50	652.00	1.22	35.90	43.62
	Female	42.40	192.33	0.97	21.33	20.69	638.00	1.25	35.03	43.83
SEM		0.32	6.35	0.03	0.90	0.99	10.16	0.05	0.60	1.77
Main effect										
NE/Lys	I (8.93)	41.85	199.00	0.92	22.45	20.53	696.00 ^A^	1.18	38.48 ^A^	45.27
	II (5.93)	41.93	193.83	0.96	21.70	20.82	656.33 ^B^	1.26	36.14 ^B^	45.55
	III (9.76)	42.38	195.67	0.96	21.90	21.09	691.67 ^A^	1.16	38.19 ^A^	44.34
	IV (6.50)	42.09	194.00	0.95	21.70	20.60	645.00 ^B^	1.23	35.47 ^B^	43.72
Sex	Male	41.99	196.58	0.95	22.08	20.92	680.25 ^a^	1.20	37.54 ^a^	45.13
	Female	42.14	194.67	0.95	21.79	20.60	664.25 ^b^	1.21	36.59 ^b^	44.32
*p*-value										
NE/Lys		0.395	0.835	0.361	0.818	0.941	<0.001	0.225	<0.001	0.720
Sex		0.510	0.675	0.968	0.652	0.653	0.041	0.756	0.039	0.526
NE/Lys × Sex		0.173	0.949	0.357	0.943	0.916	0.924	0.425	0.944	0.263

Notes: NE: net energy, IABW: Initial average body weight, FABW: Final average body weight, FCR: Feed conversion ratio, ADGW: Average daily gain weight, ADFI: Average daily feed intake. ^a,b^ means within rows with different superscripts are signiffcantly different (*p* < 0.05), ^A,B^ means within rows with different superscripts are highly signiffcantly different (*p* < 0.01). The following table is the same.

**Table 4:** Effects of net energy/lysine and sex on intestinal development of broilers at d 7 and 17.

NE/Lys	Sex	1–7 d					1–17 d				
		Glandular g/kg	Gizzard g/kg	Duodenum /cm	Jejunum /cm	Ileum /cm	Glandular g/kg	Gizzard g/kg	Duodenum /cm	Jejunum /cm	Ileum /cm
I (8.93)	Male	6.74	26.21	19.68	41.97	38.52	5.47	21.48	26.58	63.50	64.50
	Female	6.41	27.25	20.00	46.08	45.12	5.50	23.78	24.42	60.25	57.50
II (5.93)	Male	7.31	25.71	19.80	45.00	42.18	5.74	23.59	24.83	55.33	57.25
	Female	6.55	27.30	22.00	47.17	46.00	5.79	24.35	22.42	52.33	49.50
III (9.76)	Male	7.59	26.74	19.67	43.42	40.67	5.21	23.93	25.75	60.33	61.83
	Female	7.32	26.89	20.25	45.92	44.50	5.34	23.03	23.92	58.42	55.58
IV (6.50)	Male	7.23	28.06	18.47	42.72	40.92	6.08	24.09	24.00	54.85	55.18
	Female	7.67	27.78	20.67	44.25	42.67	6.28	25.76	23.25	59.92	57.17
SEM		0.14	0.45	0.30	0.60	0.51	0.10	0.33	0.29	0.86	0.91
Main effect											
NE/Lys	I (8.93)	6.58	26.73	19.84	44.03	41.82	5.49 ^b^	22.63	25.50	61.88 ^a^	61.00 ^a^
	II (5.93)	6.93	26.50	20.90	46.08	44.09	5.77 ^ab^	23.97	23.63	53.83 ^b^	53.38 ^b^
	III (9.76)	7.46	26.81	19.96	44.67	42.58	5.28 ^b^	23.48	24.83	59.38 ^a^	58.71 ^a^
	IV (6.50)	7.45	27.92	19.57	43.48	41.79	6.18 ^a^	24.93	23.63	57.38 ^b^	56.18 ^b^
Sex	Male	7.22	26.68	19.40 ^b^	43.28 ^b^	40.57 ^B^	5.62	23.27	25.29 ^A^	58.50	59.69 ^a^
	Female	6.99	27.30	20.73 ^a^	45.85 ^a^	44.57 ^A^	5.73	24.23	23.50 ^B^	57.73	54.94 ^b^
*p*-value											
NE/Lys		0.109	0.691	0.434	0.472	0.37	0.027	0.134	0.085	0.03	0.049
Sex		0.421	0.496	0.039	0.046	0.001	0.601	0.161	0.006	0.66	0.019
NE/Lys × Sex		0.519	0.88	0.543	0.887	0.438	0.987	0.369	0.747	0.31	0.239

^a,b^ means within rows with different superscripts are signiffcantly different (*p* < 0.05), ^A,B^ means within rows with different superscripts are highly signiffcantly different (*p* < 0.01). The following table is the same.

**Table 5:** Effects of net energy/lysine and sex on the intestinal morphology of broilers at d 7 and 17.

NE/Lys	Sex	1–7 d			1–17 d		
		VH/μm	CD/μm	VH/CD	VH/μm	CD/μm	VH/CD
I (8.93)	Male	632.63	127.73	4.97	541.21	147.19	3.68
	Female	787.52	187.32	4.21	834.63	141.79	5.89
II (5.93)	Male	567.26	212.10	2.76	828.73	164.31	5.05
	Female	702.71	142.38	4.94	743.67	204.70	3.72
III (9.76)	Male	709.70	193.27	3.67	602.97	180.38	3.34
	Female	829.24	140.48	5.90	833.64	125.83	6.63
IV (6.50)	Male	653.93	136.94	4.78	808.60	192.86	4.19
	Female	660.26	134.20	4.92	826.54	143.33	5.77
SEM		48.09	13.79	0.36	33.01	13.36	0.37
Main effect							
NE/Lys	I (8.93)	710.07	157.53	4.59	687.92 ^d^	144.49	4.79 ^ab^
	II (5.93)	634.99	177.24	3.85	786.20 ^ab^	184.51	4.38 ^c^
	III (9.76)	769.47	166.88	4.79	718.31 ^c^	153.11	4.99 ^a^
	IV (6.50)	657.09	135.57	4.85	817.57 ^a^	168.10	4.98 ^a^
Sex	Male	640.88	167.51	4.04 ^b^	695.38 ^b^	171.19	4.07 ^b^
	Female	744.93	151.10	4.99 ^a^	809.62 ^a^	153.91	5.50 ^a^
*p*-value							
NE/Lys		0.281	0.231	0.240	0.111	0.187	0.48
Sex		0.062	0.245	0.040	0.022	0.224	0.024
NE/Lys × Sex		0.604	0.041	0.059	0.032	0.091	0.048

^a–d^ means within rows with different superscripts are signiffcantly different (*p* < 0.05).

**Table 6:** Effects of net energy/lysine and sex on the cecal microbiota of broilers at d 7.

Items									SEM	Main Effect						***p*** **-Value**		
NE/Lys	I (8.93)		II (5.93)		III (9.76)		IV (6.50)			NE/Lys				Sex		NE/Lys	Sex	NE/Lys ×Sex
Sex	Male	Female	Male	Female	Male	Female	Male	Female		I (8.93)	II (5.93)	III (9.76)	IV (6.50)	**Male**	**Female**			
Firmicutes_A	0.742	0.792	0.699	0.724	0.524	0.646	0.646	0.686	0.035	0.767	0.711	0.585	0.666	0.653	0.712	0.346	0.413	0.962
Bacteroidota	0.067	0.028	0.132	0.11	0.182	0.242	0.164	0.098	0.042	0.047	0.121	0.212	0.131	0.136	0.119	0.598	0.839	0.956
Firmicutes_D	0.135	0.064	0.083	0.063	0.118	0.059	0.075	0.094	0.013	0.099	0.073	0.089	0.084	0.103	0.07	0.911	0.224	0.61
Proteobacteria	0.035	0.051	0.075	0.043	0.158	0.002	0.104	0.075	0.016	0.043	0.059	0.08	0.089	0.093	0.043	0.747	0.143	0.317
Actinobacteriota	0.014	0.058	0.002	0.022	0.006	0.022	0.003	0.007	0.007	0.036	0.012	0.014	0.005	0.006	0.027	0.516	0.181	0.819
Firmicutes_C	4.62 × 10^−5^	4.74 × 10^−5^	−5.42 × 10^−20^	−5.42 × 10^−20^	0	0.001	−5.42 × 10^−20^	5.81 × 10^−5^	0	4.68 × 10^−5^	−5.42 × 10^−20^	0.001	2.90 × 10^−5^	1.16 × 10^−5^	0	0.45	0.313	0.431
Campylobacterota	−6.94 × 10^−18^	0	0	0.022	1.62 × 10^−5^	0.023	4.39 × 10^−5^	0.032	0.005	0	0.011	0.012	0.016	1.50 × 10^−5^	0.019	0.726	0.077	0.727
Cyanobacteria	0	0	6.91 × 10^−5^	0.006	1.78 × 10^−5^	0	0	6.13 × 10^−5^	0.001	0	0.003	8.44 × 10^−5^	0	0	0.002	0.439	0.357	0.417
Firmicutes_B	0	0	1.75 × 10^−5^	9.14 × 10^−5^	6.99 × 10^−5^	0.001	5.23 × 10^−5^	0	0	0	5.45 × 10^−5^	0	0	3.49 × 10^−5 a^	0 ^b^	0.216	0.017	0.305
Acidobacteriota	2.31 × 10^−5^	0	7.09 × 10^−5^	5.87 × 10^−5^	4.85 × 10^−5^	2.08 × 10^−5^	6.66 × 10^−5^	1.75 × 10^−5^	0	1.16 × 10^−5^	6.48 × 10^−5^	3.46 × 10^−5^	4.21 × 10^−5^	5.23 × 10^−5^	2.43 × 10^−5^	0.519	0.275	0.96
Desulfobacterota_I	0	−6.78 × 10^−21^	−1.06 × 10^−21^	−6.78 × 10^−21^	4.66 × 10^−21^	0	4.04 × 10^−5^	3.91 × 10^−5^	0	6.03 × 10^−5^	−3.92 × 10^−21^	2.33 × 10^−21^	3.97 × 10^−5^	4.03 × 10^−5^	9.78 × 10^−6^	0.146	0.165	0.146
Spirochaetota	7.71 × 10^−5^	6.78 × 10^−21^	−3.39 × 10^−21^	0	0	6.78 × 10^−21^	6.78 × 10^−21^	2.88 × 10^−5^	0	3.85 × 10^−5^	−1.69 × 10^−21^	0	1.44 × 10^−5^	1.93 × 10^−5^	7.19 × 10^−6^	0.137	0.347	0.047
Chloroflexota	−1.69 × 10^−21^	−1.69 × 10^−21^	0	0	6.78 × 10^−21^	3.21 × 10^−5^	0	−3.39 × 10^−21^	0	−1.69 × 10^−21^	0	1.61 × 10^−5^	−1.69 × 10^−21^	−1.69 × 10^−21^	8.04 × 10^−6^	0.418	0.332	0.418
Gemmatimonadota	0	0	1.75 × 10^−5^	0	0	3.11 × 10^−5^	0	3.39 × 10^−21^	0	0	8.74 × 10^−6^	1.56 × 10^−5^	1.69 × 10^−21^	4.37 × 10^−6^	7.79 × 10^−6^	0.557	0.707	0.314
Verrucomicrobiota	2.12 × 10^−21^	1.99 × 10^−5^	0	8.47 × 10^−22^	2.67 × 10^−5^	−4.24 × 10^−22^	3.51 × 10^−5^	−4.66 × 10^−21^	0	9.96 × 10^−6^	0	1.34 × 10^−5^	1.75 × 10^−5^	1.55 × 10^−6^	4.98 × 10^−6^	0.767	0.399	0.384
Desulfobacterota_B	4.24 × 10^−22^	4.24 × 10-22	3.95 × 10^−5^	3.05 × 10^−5^	4.24 × 10^−22^	4.24 × 10^−22^	2.12 × 10^−21^	−1.27 × 10^−21^	0	4.24 × 10^−22^	3.50 × 10^−5^	4.24 × 10^−22^	4.24 × 10^−22^	9.88 × 10^−6^	7.62 × 10^−6^	0.16	1	1
Nitrospirota_A	1.69 × 10^−21^	0	0	1.69 × 10^−21^	0	3.39 × 10^−21^	2.29 × 10^−05^	−1.69 × 10^−21^	0	0	0	1.69 × 10^−21^	1.15 × 10^−5^	5.73 × 10^−6^	8.47 × 10^−22^	0.418	0.332	0.418

^a,b^ means within rows with different superscripts are signiffcantly different (*p* < 0.05).

**Table 7:** Effects of net energy/lysine and sex on the cecal microbiota of broilers at d 17.

Items									SEM	Main Effect						*p*-Value		
NE/Lys	I (8.93)		II (5.93)		III (9.76)		IV (6.50)			NE/Lys				Sex		NE/Lys	Sex	NE/Lys × Sex
Sex	Male	Female	Male	Female	Male	Female	Male	Female		I (8.93)	II (5.93)	III (9.76)	IV (6.50)	Male	Female			
Firmicutes_A	0.435	0.506	0.547	0.734	0.541	0.349	0.608	0.55	0.046	0.471	0.641	0.445	0.579	0.533	0.535	0.428	0.982	0.526
Bacteroidota	0.4	0.293	0.359	0.036	0.327	0.409	0.292	0.367	0.043	0.347	0.197	0.368	0.329	0.344	0.276	0.513	0.442	0.33
Firmicutes_D	0.055	0.046	0.067	0.061	0.049	0.049	0.073	0.053	0.009	0.05	0.064	0.049	0.063	0.061	0.052	0.889	0.627	0.982
Proteobacteria	0.008	0.032	0.008	0.006	0.037	0.063	0.013	0.002	0.009	0.02	0.007	0.05	0.007	0.017	0.026	0.295	0.622	0.832
Actinobacteriota	0.011	0.107	0.007	0.103	0.024	0.015	0.003	0.01	0.016	0.059	0.055	0.02	0.007	0.011	0.059	0.596	0.157	0.529
Firmicutes_C	0.08	5.811 × 10^−5^	0	1.00 × 10^−3^	2.772 × 10^−5^	0.101	0	0	0.012	0.04	0	0.05	0	0.02	0.025	0.353	0.83	0.114
Campylobacterota	3.00 × 10^−3^	0.004	0.003	0.022	0.003	0.003	0	0.006	0.003	0.004	0.013	0.003	0.003	0.002	0.009	0.581	0.282	0.672
Cyanobacteria	0	0.001	0.001	0.031	0.007	0.003	0.004	0.005	0.003	0	0.016	0.005	0.005	0.003	0.01	0.417	0.316	0.28
Firmicutes_B	0.001	0.001	0	0.002	0	0	0	0.001	0	0.001	0.001	0	0.001	0	0.001	0.714	0.387	0.378
Acidobacteriota	5.168 × 10^−5^	0	1.977 × 10^−5^	3.590 × 10^−5^	0	3.900 × 10^−5^	5.499 × 10^−5^	5.106 × 10^−5^	0	2.584 × 10^−5^	2.783 × 10^−5^	1.950 × 10^−5^	5.303 × 10^−5^	3.161 × 10^−5^	3.149 × 10^−5^	0.6	1	0.38
Desulfobacterota_I	0	2.91 × 10^−5^	2.97 × 10^−5^	0	1.694 × 10^−21^	3.452 × 10^−5^	0	4.204 × 10^−5^	0	1.453 × 10^−5^	1.48 × 10^−5^	1.726 × 10^−5^	2.102 × 10^−5^	7.413 × 10^−6^	2.640 × 10^−5^	0.992	0.283	0.452
Spirochaetota	2.878 × 10^−5^	3.874 × 10^−5^	1.27 × 10^−21^	1.271 × 10^−21^	1.386 × 10^−5^	8.470 × 10^−22^	8.470 × 10^−22^	−2.54 × 10^−21^	0	3.376 × 10^−5^	1.27 × 10^−21^	6.931 × 10^−6^	−8.47 × 10^−22^	1.066 × 10^−5^	9.684 × 10^−6^	0.22	1	0.927
Chloroflexota	0	0	0	5.983 × 10^−5^	−6.78 × 10^−21^	3.900 × 10^−5^	−1.69 × 10^−21^	1.69 × 10^−21^	0	0	2.992 × 10^−5^	1.950 × 10^−5^	0	1.694 × 10^−21^	2.471 × 10^−5^	0.467	0.137	0.467
Gemmatimonadota	−2.47 × 10^−21^	2.905 × 10^−5^	2.792 × 10^−5^	2.052 × 10^−5^	7.764 × 10^−22^	−2.12 × 10^−22^	−2.61 × 10^−21^	7.694 × 10^−21^	0	1.453 × 10^−5^	2.422 × 10^−5^	2.823 × 10^−22^	2.541 × 10^−21^	6.980 × 10^−6^	1.239 × 10^−5^	0.377	0.639	0.681
Verrucomicrobiota	0	2.142 × 10^−5^	0	0	0	0	0	0	0	1.071 × 10^−5^	0	0	0	0	5.354 × 10^−6^	0.418	0.332	0.418
Firmicutes_G	0	0	0	0	7.855 × 10^−5^	0	−6.78 × 10^−21^	0	0	0	0	3.927 × 10^−5^	−3.39 × 10^−21^	1.964 × 10^−5^	0	0.418	0.332	0.418
Myxococcota_A	−3.39 × 10^−21^	1.937 × 10^−5^	−3.39 × 10^−21^	−3.39 × 10-21	−3.39 × 10^−21^	2.878 × 10^−5^	−3.39 × 10^−21^	1.953 × 10^−5^	0	9.684 × 10^−6^	−3.39 × 10^−21^	1.439 × 10^−5^	9.767 × 10^−6^	−2.97 × 10^−21^	1.692 × 10^−5^	0.776	0.108	0.776
Nitrospirota_A	0	0	0	0	0	2.589 × 10^−5^	0	9.49 × 10^−6^	0	0	0	1.295 × 10^−5^	4.745 × 10^−6^	8.470 × 10^−22^	8.846 × 10^−6^	0.52	0.218	0.52
Desulfobacterota_G	0	0	0	4.787 × 10^−5^	0	0	0	0	0	0	2.393 × 10^−5^	0	0	0	1.197 × 10^−5^	0.418	0.332	0.418

## Data Availability

The original contributions presented in the study are included in the article; further inquiries can be directed to the first author.
